# Continuous Magnitude Production of Loudness

**DOI:** 10.3389/fpsyg.2021.635557

**Published:** 2021-05-11

**Authors:** Josef Schlittenlacher, Wolfgang Ellermeier

**Affiliations:** ^1^Manchester Centre for Audiology and Deafness, Division of Human Communication, Development and Hearing, School of Health Sciences, University of Manchester, Manchester, United Kingdom; ^2^Applied Cognitive Psychology Unit, Department of Psychology, Technische Universität Darmstadt, Darmstadt, Germany

**Keywords:** loudness, time-varying, methods, cross-modality matching, line length, magnitude production

## Abstract

Continuous magnitude estimation and continuous cross-modality matching with line length can efficiently track the momentary loudness of time-varying sounds in behavioural experiments. These methods are known to be prone to systematic biases but may be checked for consistency using their counterpart, magnitude production. Thus, in Experiment 1, we performed such an evaluation for time-varying sounds. Twenty participants produced continuous cross-modality matches to assess the momentary loudness of fourteen songs by continuously adjusting the length of a line. In Experiment 2, the resulting temporal line length profile for each excerpt was played back like a video together with the given song and participants were asked to continuously adjust the volume to match the momentary line length. The recorded temporal line length profile, however, was manipulated for segments with durations between 7 to 12 s by eight factors between 0.5 and 2, corresponding to expected differences in adjusted level of −10, −6, −3, −1, 1, 3, 6, and 10 dB according to Stevens’s power law for loudness. The average adjustments 5 s after the onset of the change were −3.3, −2.4, −1.0, −0.2, 0.2, 1.4, 2.4, and 4.4 dB. Smaller adjustments than predicted by the power law are in line with magnitude-production results by Stevens and co-workers due to “regression effects.” Continuous cross-modality matches of line length turned out to be consistent with current loudness models, and by passing the consistency check with cross-modal productions, demonstrate that the method is suited to track the momentary loudness of time-varying sounds.

## Introduction

There are numerous methods for the subjective evaluation of auditory stimuli for a variety of purposes. Building upon Fechner’s (1860) seminal work describing the three classical methods of threshold measurement, and proposing a rationale for psychophysical scale construction based on just-noticeable differences, transformed up-down methods (Levitt, 1971) have become the gold standard both for determining discriminability, and for adjusting two stimuli to equal sensation. Transformed up-down methods are subject to fewer biases than the classical methods because the task for the participant is rather simple. When evaluating loudness, the question is typically “Which of the two sounds was louder?,” and the level of the target stimulus is adjusted before the next presentation of the pair. Disadvantages of this method are that it needs a reference, that it can only be applied to measure thresholds or points of subjective equality, and that it is time-consuming because determining a point of subjective equality requires several trials.

In contrast, magnitude estimation (Stevens, 1956, 1957, 1975) does not require a reference, can easily cover a large range of stimulus intensities, and yields one estimate of a psychophysical scale value per trial. However, it is prone to biases because the task of scaling is left to the participant (Luce and Mo, 1965; Luce and Krumhansl, 1988). Some of these biases have been extensively studied in the framework of direct magnitude scaling (e.g., Stevens and Galanter, 1957; Teghtsoonian and Teghtsoonian, 1978; Poulton, 1979; DeCarlo and Cross, 1990). Others have been conceptualized within the framework of axiomatic measurement (Narens, 1996; Ellermeier and Faulhammer, 2000; Luce, 2002; Zimmer, 2005) or even Bayesian inference (Petzschner et al., 2015).

A basic check for the consistency of direct scaling outcomes has frequently been to perform magnitude production, which can be seen as the inverse procedure of magnitude estimation (Reynolds and Stevens, 1960): Instead of rating the magnitude of a stimulus, the stimulus is adjusted to match a given estimate. Magnitude production typically yields larger exponents than magnitude estimation, i.e., a smaller level change is needed to e.g., double loudness than magnitude estimates would suggest. Stevens and Greenbaum (1966) explained this phenomenon by “regression effects,” which occur whenever two continua are matched in both directions because participants compress the range of the variable that they adjust.

A further opportunity to verify the consistency of scaling procedures is provided by the method of cross-modality matching. A very straightforward case is matching a given sensation with a line length to be produced: Instead of assigning a number to the magnitude of the stimulus, the length of a line is adjusted to match the subjective magnitude (Stevens and Galanter, 1957; Stevens and Guirao, 1963). Stevens and Greenbaum (1966) highlight the similarities between the matching and scaling methodologies by interpreting magnitude estimation as an “instance of the general method of cross-modality matching” (p. 441) to the number continuum.

Cross-modality matching with line length has also been used for continuous judgment of loudness (see Kuwano, 1996, and Kuwano and Namba, 2011, for an overview). Continuous judgment allows us to obtain estimates for the momentary loudness of time-varying sounds, where trial-based methods can only give estimates of the overall loudness of the segments that were presented. Continuous judgment may also be used with the goal to maximize the number of estimates that are obtained per experiment time, somewhat similar to Békésy tracking for obtaining thresholds (von Békésy, 1947).

Continuous judgment of auditory sensations, most commonly loudness, was first done using categories (Namba and Kuwano, 1980; Kuwano and Namba, 1985), with the participants pressing the button for the current category on a response box. Alternative methods used the position of a slider (Fastl, 1991) or cross-modality matching with a muscular force by employing a lever with force feedback (Susini et al., 2002). Several studies used continuous cross-modality matching with line length to track momentary loudness or similar auditory magnitudes, where typically the length of a line that is displayed on a computer screen can be modified by moving the mouse (e.g., Namba and Kuwano, 1990; Kuwano et al., 2003; Kuwano et al., 2014, 2017; Schlittenlacher et al., 2017).

To our knowledge, the methodology of continuous judgment lacks thorough investigation and consistency checks like the ones that have been performed for conventional magnitude estimation or cross-modality matching. To evaluate the consistency of continuous judgment, in Experiment 1, we had participants make continuous cross-modality matches of line length in response to temporally varying loudness patterns of musical songs. In Experiment 2, we inverted the procedure by having participants generate continuous magnitude productions of loudness in response to lines dynamically changing in length. We also analysed the temporal portion on which momentary line length matches are based. In contrast to Kuwano and Namba (1985), we did not use temporal windows with hard cutoffs but varied the exponential time constant in a loudness model (Moore et al., 2018) to find the highest correlation with the momentary line length matches and to evaluate the choice made by the loudness model.

## Materials and Methods

All participants completed two experiments involving cross-modality matches between loudness and line length. In the first experiment, they continuously adjusted the length of a line to match their impression of loudness. In the second experiment, they performed the reverse operation, i.e., they made magnitude productions of loudness by continuously adjusting sound levels so that their loudness matched dynamically changing line lengths that were displayed simultaneously with the sounds.

### Participants

Twenty listeners, eight females and twelve males, participated in the experiments. They were aged 18 to 50 years, with a median age of 23 years. All of them participated in both experiments. Their hearing sensitivity was better than 20 dB HL at each frequency between 125 and 8,000 Hz at both ears. They participated voluntarily without compensation after having given informed consent.

### Apparatus

The auditory stimuli were stored as wav files, D/A converted by an RME Hammerfall DSP Multiface II audio interface (Haimhausen, Germany) and presented via Sennheiser HDA 200 headphones (Wedemark, Germany). The participants sat in a double-walled sound-proof booth (IAC, Chandler’s Ford, Hampshire, United Kingdom).

Calibration was done according to Richter (2003): The sound pressure level of a 1-kHz tone was measured in a Bruel & Kjaer 4153 coupler with DB-0843 adapter plate. To obtain a free-field level rather than coupler measurement, the difference of −3.5 dB between coupler sensitivity and free-field sensitivity (Table 3.1.3 in Richter, 2003) was added.

The participants used a computer mouse for making their responses. In experiment 1, movement of the mouse to the left or right changed the length of a line that was displayed horizontally on a screen. In experiment 2, the mouse was used to press buttons on the screen. The horizontal screen resolution was 1,280 pixels (px). Line lengths and button presses were recorded using the internal clock of Microsoft Windows XP, which has a rate of 16 ms. For statistical analyses and further processing, line lengths or adjusted levels between the timestamps were upsampled to a rate of 1 ms by linear interpolation.

### Stimuli

The stimuli were fourteen excerpts of musical pieces with durations between 142 and 251 s. Their combined duration amounted to 45 min. Seven of the excerpts were from the rock genre and seven were selected from classical music. The distinction between genres was made to have stimuli with little variations in loudness over time (i.e., rock music excerpts) and other ones having a large dynamic range (i.e., the classical music samples). For each excerpt, the two tracks of the stereo file were merged for diotic presentation. The levels were adjusted so that the seven songs in each genre had overall calculated loudness levels (DIN 45631/A1, 2010, N5) of 70, 74, 78, 82, 86, 90, and 94 phon, respectively.

### Procedure of Experiment 1

The participants were instructed to continuously adjust the length of a line to match the momentary loudness of the musical excerpt while it was being played: “Please adjust the length of the line by moving the mouse so that it matches your impression of loudness at any time.” When participants asked for clarification of “at any time” (German: “zu jeder Zeit”), they were told that it was up to them to define “at any time,” and they could form that opinion during three practice trials. The line was depicted horizontally, starting on the left of the screen, having a height of 2 px, and a maximum length of 1,260 px. At the start of a trial, its length was set to 10 px so that a line was clearly visible. The length of the line could be adjusted by moving the mouse. After a song finished, there was a silent interval of 3 s after which the participants were asked to adjust the length of the line to the perceived overall loudness of the sound that they had just heard. After this they could take a break or start the next song.

Before commencing with the fourteen songs, the participants went through a short practice consisting of three stimuli which were 20-s long segments of music with a calculated overall loudness of 70, 80, and 90 phon, respectively. After this practice, participants were told that these sounds represented the loudness range to be expected during the main experiment, so that they could “recalibrate” their line length. No reference line length was given. Participants were allowed to repeat the practice.

### Procedure of Experiment 2

Experiment 2 took place right after Experiment 1. The participants were encouraged to take a break for as long as they wanted.

For Experiment 2, the participants were asked to continuously adjust the loudness of the sound to match the line length that they saw on the screen (displayed as in Experiment 1): “Please use the + and – buttons to adjust the loudness so that it matches the length of the line at any time.” The lines were shown like in a video while the songs were being played. The level could be adjusted by using plus and minus buttons, each of which changed the level by 1 dB per click. The participants saw their individual line length sequences that they had produced during Experiment 1, with some critical manipulations, as specified in the next paragraph. That way, they did not experience a perfect covariation between line length and loudness, but rather had to react to make them match.

There were eight manipulations per sound, and each manipulation increased or decreased the line length for a segment of between 7 and 12 s duration. The magnitude of the manipulations corresponded to −10, −6, −3, −1, +1, +3, +6, and +10 dB according to Stevens’s power law, i.e., the line length was multiplied by 0.5, 0.66, 0.81, 0.93 1.07, 1.23, 1.51, or 2.0, respectively. This implies exponents of 0.6 for sound pressure level and 1 for line length. These “line length gains” as we might call them were constant factors by which the time-varying line lengths were multiplied for the duration of the manipulation. They were introduced smoothly with linear rise and fall times of 500 ms before fully reaching the respective factor, i.e., the factor changed smoothly between 1 and the target factor.

An individual latency constant was derived from Experiment 1 and subtracted from the temporal position in the musical track, in order to subjectively align the line lengths displayed with the temporal segments of the songs they referred to. This latency constant was determined to be the offset that resulted in the highest correlation between adjusted line length and calculated momentary loudness (DIN 45631/A1). We assume that this latency covers the reaction time to changes in loudness and the time that is needed to handle the mouse. This was done for each participant and each sound.

In summary, the participants listened to a stimulus whose loudness varied over time (the music) and saw a line varying in length accordingly (the one that they produced in Experiment 1). This is different from traditional magnitude production where the participants make adjustments to a stationary stimulus. During eight intervals in each song, however, the line length displayed was manipulated and the participants were supposed to adjust the loudness after onset and offset of these manipulations.

## Results

Before comparing the cross-modality matching results of Experiment 1 to calculated loudness, we look at the magnitude productions made in Experiment 2.

### Results of Experiment 2: Matching Sound Levels to Line Lengths

Table 1 shows the adjustments in sound level that were made 5 s after the onset of a line-length gain compared to the level 2 s before it started. 5 s were chosen because we think that this is long enough to account for any delay in a participant’s reaction, and still within the 7 to 12-s window of the manipulation. These changes range from −3.3 dB for halving the line length to +4.4 dB for doubling it, which is considerably less than what would be expected from Stevens’s power law. However, the changes in level made in response to the artificial line-length gains significantly differ from zero except for the two smallest manipulations in line lengths (factors of 0.93 and 1.07), according to *t*-tests which were calculated independently for each gain factor (last two columns of Table 1).

**TABLE 1:** Adjustment in level in response to the onset of the line length manipulations given in the first column.

**Line length factor**	**Mean change [dB]**	**SD [dB]**	***t*-value**	***p*-value**
0.5	–3.3	2.9	–17.4	< 0.001
0.66	–2.4	2.9	–13.2	< 0.001
0.81	–1.0	2.4	–6.5	< 0.001
0.93	–0.2	3.1	–1.1	0.3
1.07	0.2	2.2	1.3	0.2
1.23	1.4	3.3	6.5	< 0.001
1.51	2.4	3.7	10.6	< 0.001
2	4.4	5.1	14.2	< 0.001

*Means and standard deviations across subjects and stimuli, and one-sample *t*-tests comparing to zero change.*

The opposite pattern in level adjustment would be expected after the offset of the manipulations in line length, i.e., after cancelling the artificial line-length gains and returning to the baseline pattern produced in Experiment 1. Table 2 shows the adjustments that were made 5 s after the end of a manipulation in line length compared to the level 2 s before the end of a manipulation. They range from +2.8 to −3.9 dB, and all of them differ statistically significantly from zero. The sum of the mean values in Tables 1, 2 ranges from −0.5 to 0.5 dB and is 0.0 dB on average, which indicates that on average, the level adjustment to the onset of a manipulation was reversed after its offset.

**TABLE 2:** Adjustment in level to the offset of line length manipulations in the first column.

**Line length factor**	**Mean change [dB]**	**SD [dB]**	***t*-value**	***p*-value**
0.5	2.8	2.9	15.2	< 0.001
0.66	1.9	3.1	10	< 0.001
0.81	1.4	2.8	8.2	< 0.001
0.93	0.4	2.4	2.7	< 0.01
1.07	–0.3	1.8	–2.7	< 0.01
1.23	–1.2	3.1	–6.2	< 0.001
1.51	–2.7	3.4	–12.6	< 0.001
2	–3.9	3.9	–16.6	< 0.001

*Means and standard deviations across subjects and stimuli, and *t*-tests comparing to zero.*

In contrast to a classical magnitude production experiment, where one production is made per trial, participants may “fall asleep,” lose track, and not make any adjustment. Table 3 lists the percentages of adjustments in the correct direction (an increase of at least 1 dB when the line length increased or a decrease of at least 1 dB when the line length decreased), no change in level, or a change in the wrong direction. The largest line length gains led change of level in the correct direction in 78% of all cases. The two smallest gain factors produced no change in 40 or 45%, respectively, and 33% changes in the correct direction. For all of the gain factor manipulations, more changes were made in the expected direction than in the opposite.

**TABLE 3:** Type of level adjustment in response to the stimulus manipulations given in the first column.

**Line length factor**	**Correct direction [%]**	**No change [%]**	**Wrong direction [%]**
0.5	78	18	4
0.66	69	22	9
0.81	48	38	14
0.93	33	40	26
1.07	33	45	21
1.23	59	27	13
1.51	68	21	10
2	78	13	10

**FIGURE 1:**
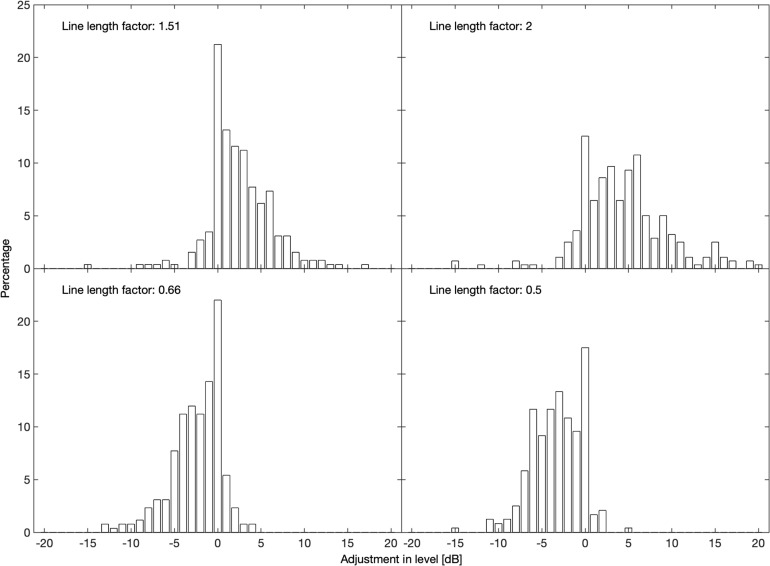
Distributions of level adjustments 5 s after the onset of a manipulation in line length for factors in line length of 1.51 (upper left), 2 (upper right), 0.66 (lower left), and 0.5 (lower right).

Figure 1 shows the distributions of the adjustments in level 5 s after onset in 1-dB wide bins for the four largest gain factors (0.5, 0.66, 1.51, and 2.0), which were summarized in Table 1. For all four of them, only a small fraction reaches or exceeds the adjustment that would be expected according to Stevens’s power law, i.e., of −10, −6, 6, and 10 dB, respectively. Each of them shows a peak at 0 dB, i.e., when no adjustment was made (as in Table 3). All distributions drop sharply on the “wrong” side of 0 dB.

One may speculate whether the continuous magnitude productions differ for the two music genres since rock songs have more uniform levels than classical music. Table 4 shows the adjustments to the manipulations in sound level separately for each music genre (otherwise the same as the means in Tables 1, 2). The level adjustments are rather similar for the two genres, except for the line length factor of 2 where the adjustments for rock music were about 1 dB larger than those for classical music. To test this discrepancy for statistical significance, we performed a three-way within-subjects analysis of variance with factors line length factor (−0.5 to 2), genre (rock, classic) and direction (onset, offset). The main effect for the line length factor was highly significant, *F*(7,133) = 118, *p* < 0.001. The main effect for genre was not statistically significant, *F*(1,19) = 3.7, *p* = 0.07, neither was the main effect for direction, *F*(1,19) = 0.01, *p* = 0.92. Most critically for the observed difference, the interaction between the line length factor and genre was statistically significant, *F*(7,133) = 2.4, *p* < 0.05. The interactions between line length factor and direction, *F*(7,133) = 3.4, *p* < 0.01, and between genre and direction, *F*(1,19) = 18, *p* < 0.001 were also statistically significant. The three-way interaction was not statistically significant, *F*(7,133) = 1.7, *p* = 0.11.

**TABLE 4:** Adjustment in level [dB] to the onset and offset of the line length manipulations by music genre.

	**Classic music**		**Rock music**	
**Line length factor**	**Onset**	**Offset**	**Onset**	**Offset**
0.5	–3.5	2.4	–3.1	3.1
0.66	–2.6	1.4	–2.3	2.3
0.81	–1.0	1.4	–1.0	1.5
0.93	–0.4	0.1	0.0	0.7
1.07	0.1	–0.3	0.3	–0.3
1.23	0.9	–1.1	1.9	–1.4
1.51	2.6	–2.6	2.3	–2.8
2	3.5	–3.6	5.2	–4.2

*Same as second columns (means) of Tables 1, 2 but separately for each genre.*

### Results of Experiment 1: Continuous Matching of Line Length to Sound Levels

Experiment 1 was analysed to compare the line lengths produced via cross-modality matching with loudness calculations based on the model of Moore et al. (2018). This model produces three estimates of time-varying loudness: (1) Instantaneous loudness, which is not available to conscious perception and based on a single momentary spectrum; (2) Short-term loudness, which represents the loudness of short segments such as a syllable and calculated from instantaneous loudness using exponential time constants for attack and release in the order of a few ten milliseconds; and (3) Long-term loudness, which represents the loudness of longer segments such as a word or a sentence and is obtained from short-term loudness via exponential time constants, 100 ms for attack and 750 ms for release.

**FIGURE 2:**
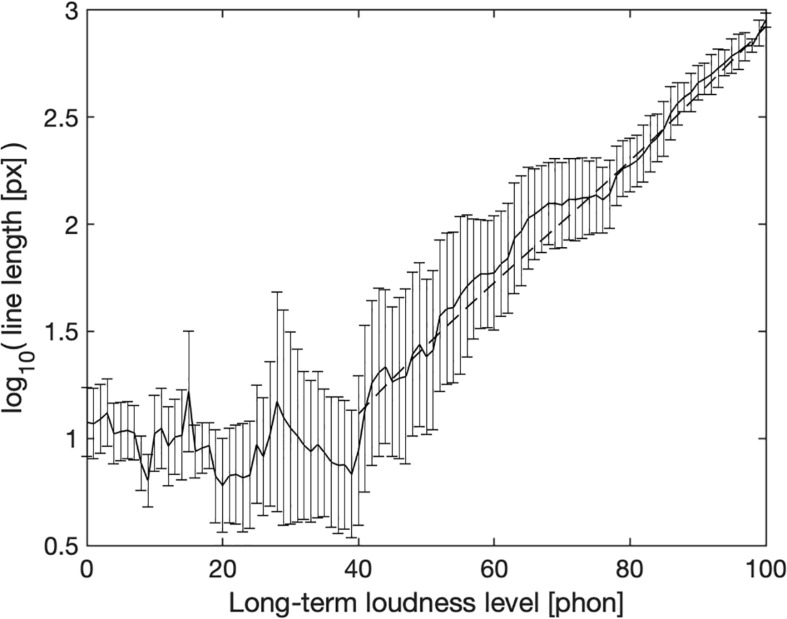
Logarithmic line length as a function of momentary long-term loudness level in 1-phon wide bins. The solid line shows averages across participants and temporal segments (and thus stimuli), error bars ± 1 standard deviation across time. The dashed line shows a correspondence of 1 sone to 13 pixels.

Figure 2 shows mean logarithmic line length as a function of calculated long-term loudness level (thick black line). Error bars represent the standard deviation across points in time that fell within a 1-phone wide bin of calculated loudness level after logarithmic line lengths were averaged across subjects for each point in time. Note, that only 20 s of the total stimulus time of 45 min had loudness levels lower than 40 phon and it is probably difficult to discriminate very short line lengths, explaining the noisy function evident at these low levels, while each 1-phon-wide bin above 65 phon represents 30 to 140 s. The participants seem to have chosen a short line of about 10 px in length independently of loudness level to represent loudnesses below 40 phon. Above 40 phon, mean line length correlates highly with calculated long-term loudness, *r*(58) = 0.99, *p* < 0.001. The correlation between line length and calculated long-term loudness without averaging across time per phon bin, i.e., using the raw data points, is still high, *r*(2690225) = 0.89, *p* < 0.001. To compute this correlation, line length was upsampled to a resolution of 1 ms to match the sample rate of calculated long-term loudness. The fact that the relationship (above 40 phons) is nearly linear in log-log coordinates is evidence for an excellent fit to a power function. The dashed line shows it to imply a 13 px line-length increment for each loudness increase by 1 sone.

**FIGURE 3:**
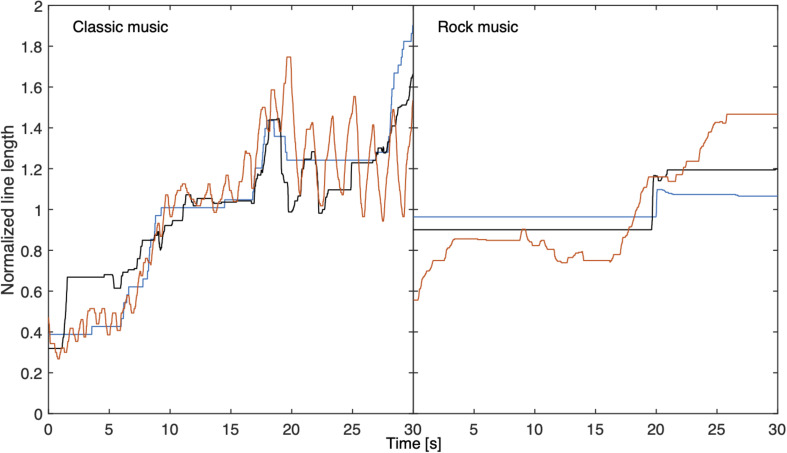
Normalized matched line lengths for three example participants for a 30-s segment of a classical piece (left) and of a rock song (right). The line length was normalized for the 30-s segment.

An important question in continuous psychophysical scaling is which temporal portions of the sound impact a momentary judgment. Figure 3 shows normalized line length matches to 30-s excerpts of a classical piece and a rock song for three illustrative participants who apparently had different strategies for making continuous line-length adjustments. The loudness model of Moore et al. (2018) uses an exponential time constant of 750 ms for long-term loudness based on time-varying binaural stimuli. The present data can also be used to estimate this time constant, although this estimate may be limited by the ability to move the mouse. For this purpose, we calculated correlation coefficients between adjusted line length and long-term loudness for each song and participant, and varied the release time constant of long-term loudness between 0 and 3,000 ms, while keeping all other time constants as suggested by the model. The latency, a delay for producing a corresponding line length, was varied between 0 and 3,000 ms. The time constant and latency that yielded the highest correlation coefficient for each song and participant were taken as the “true” values.

**FIGURE 4:**
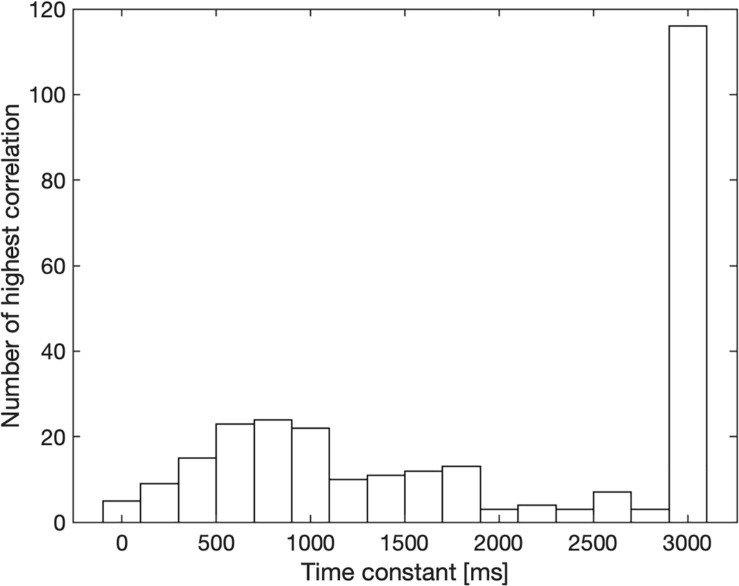
Release time constants for long-term loudness that yielded the highest correlation between line length and calculated long-term loudness for each participant and sound. The number of occurrences within 200-ms wide bins is shown on the ordinate. The search ranged from 0 to 3,000 ms.

The mean latency turned out to be 826 ms. The time constants estimated for long-term loudness are shown in Figure 4. 41% of the stimuli (classic: 31%, rock: 50%) yielded the maximum time constant of 3,000 ms, indicating that an even longer integration time may have produced a higher correlation and participants only moved the line to considerable changes in loudness. Interestingly, the distribution in Figure 4 shows a local maximum close to the model’s time constant of 750 ms. Fitting the distribution with two Gaussians, and not taking into account the time constants of 3,000 ms or longer, yielded means of 710 and 1,730 ms (averaged across 100 runs for the fit, ranges of the means: 700 to 720 ms and 1,690 to 1,760 ms).

## Discussion

In two laboratory experiments it was shown that two instances of cross-modality matching, (1) continuous matching of line length to time-varying loudness, and (2) its inverse, continuous matching of loudness to temporally varying line lengths yielded meaningful results in terms of (a) validity of responses to stimulus changes, (b) psychophysical functions, and (c) the time constants involved: (a) Participants followed the direction of the experimentally manipulated line length changes in the magnitude-production task despite those manipulations being embedded in long musical excerpts that already varied over time. (b) The exponent of the psychophysical function for continuous matching of line length agreed with predictions of a loudness model since on average, line length as a function of long-term loudness exhibited a simple linear relation between pixels and sone (dashed line in Figure 2), and was steeper in the magnitude production task due to a regression effect that was known to exist to a lesser extent for stationary stimuli. (c) The time constants exhibited a local mode at a value that was also found using a different approach based on binaural effects (Moore et al., 2018). This had not been demonstrated to that extent for stimuli continuously varying in magnitude over time.

Some peculiarities of the present results, however, deserve discussion. The exponent of loudness as a function of sound pressure is typically steeper for magnitude production than it is for magnitude estimation (Reynolds and Stevens, 1960), i.e., a difference of less than 10 dB is required to double loudness. Stevens and co-workers found exponents of 0.7, corresponding to 9 dB being required to double loudness, in magnitude-production tasks (Stevens and Guirao, 1962; Stevens and Greenbaum, 1966; **Figure 5**); Hellman (1981) reported an exponent of 0.81 (7 dB to double loudness) for a 1-kHz pure tone. Teghtsoonian and Teghtsoonian (1978) found that the exponent depended on the range of magnitudes that is presented. For a range of 0.5 log units, which corresponds to a factor of 3.2 (close to the maximal ratio of manipulations used in the present magnitude production experiment), they reported an exponent of 1.1 (i.e., 5 dB to double loudness).

The results of the present magnitude production task (Experiment 2) suggest a difference of 3 or 4 dB to match the loudness after doubling or halving line length. This is considerably less than the 10 dB that Stevens’s power law suggests, and also less than in all other studies cited. However, the range of our manipulations from 0.5 to 2 (a factor of 4) was rather narrow, for which Teghtsoonian and Teghtsoonian (1978) found results more similar to ours. Furthermore, the modes at 0 dB (i.e., no adjustment made, see Figure 1) indicate that the participants sometimes failed to track a stimulus change in the continuous task. About 50% of the adjustments to factors of 0.5 and 2 in line length had absolute values between 2 and 6 dB, confirming a mean sound level change of about 4 dB for line length changes suggesting a doubling or halving of subjective magnitude. Another contribution to the regression effect may be that participants were reluctant to change the stimulus level in the magnitude production task to the extent called for by the altered line lengths because they would be producing sounds unlike those they had heard in the estimation task.

The results of the line-length task agreed well with calculated long-term loudness (Figure 2), which suggests that they reproduced the exponent that underlies the loudness model. The loudness model predicts a doubling of loudness for an increase of 10 dB for a 1-kHz tone above 40 dB SPL. For other sounds, the amount that is needed to double loudness is slightly different, but similar. For example, a pink noise that spans from 50 to 20,000 Hz and has an overall level of 40 dB SPL needs an increase of 9 dB to double its loudness. In contrast to this, the 3 to 4 dB that were necessary to double loudness in the magnitude production task are considerably less.

The possible difference between genres deserves attention, too. There was no statistically significant main effect of genre in the magnitude production task. This was to be expected since the line length manipulations were balanced in both directions and thus the grand means are close to 0 dB for both genres. However, the interaction between line length factor and genre was statistically significant. This could suggest that the slightly higher absolute values for rock music, in particular for a line length factor of 2, were not due to chance. We want to emphasize that we did not formulate a specific hypothesis prior to this analysis between genres, which is why it should be considered exploratory.

Figure 2 shows a good correspondence between the continuously tracked line length (Experiment 1) and calculated momentary loudness, a line that relates 1 sone to 13 pixels approximates the averages well. Standard deviations decrease on a logarithmic scale of line length with increasing calculated loudness level, suggesting that the participants judged the louder parts reliably, which are the most important ones to inform judgments of overall loudness (DIN 45631/A1, 2010; Schlittenlacher et al., 2014, 2017; Moore et al., 2018). The good agreement between the line lengths of Experiment 1 and calculated momentary loudness in linear units (pixel and sone, dashed line in Figure 2) is at odds Stevens’s (1975) suggestion to average the exponents across estimation and production experiments to obtain a “balanced” estimate: The present results suggest that predictions of the loudness model agree with subjective evaluations in a line-length task.

To our knowledge the study of Kuwano and Namba (1985) has been the only one to date that analysed the time interval that is used to inform a momentary judgment. They presented a 20-min long recording of road traffic range during which A-weighted sound pressure level varied between about 50 and 90 dB(A), and depended mainly on the presence or absence of vehicles. They correlated the momentary judgment by category with the equivalent A-weighted sound pressure level and found the highest correlation for an integration time interval of 2.5 s. The analysis of the present paper did not use a time window but an exponential time constant, which is expected to produce somewhat shorter durations for the best match. Thus, the means of 710 and 1730 ms of the Gaussian mixture that represents 60% of the stimuli in the present study are broadly in line with the results of Kuwano and Namba.

The loudness model of Moore et al. (2018) uses an exponential time constant of 750 ms to calculate long-term loudness. This time constant was derived from time-varying synthetic stimuli that differed across the two ears. Figure 4 provides some support for this time constant: Time constants around 700 ms yielded the highest correlation between calculated long-term loudness and momentary line length more often than others. However, for many songs and participants a rather long time constant of 3 s or more produced the highest correlation. In these cases, the participants may have seen the line length to reflect the current setting of a volume control in which one would tolerate regular fluctuations in loudness or different loudness for different instruments. Furthermore, they may have been reluctant to follow the marginal changes in loudness of rock songs that are typically compressed to a small dynamic range. The long total duration of stimuli, 45 min, though with breaks, may have contributed to this effect. This kind of bias may also occur to a lesser extent in noise studies, where participants focus on a single noise source and not a band or orchestra. The long duration of a music piece compared to echoic memory in combination with the fact that adjustments in line length took time may be a further explanation for the long time constants found.

The mean latency of 826 ms is in the range of values found in the literature for cross-modality matching: Kuwano and Namba (1985) reported 1.0 s, Susini et al. (2002) 0.9 and 1.1 s for their two experiments, and Schlittenlacher et al. (2017) 495 ms.

Taken together, the results of Experiments 1 and 2 suggest that cross-modality matching of line length is a suitable method to assess momentary loudness. Its counterpart, continuous magnitude production of loudness in response to varying line length stimulation, largely agreed with the literature, though the level changes that were produced for a given change in magnitude were on the lower end of the expected range.

## Data Availability Statement

The data analyzed in this study are subject to the following licenses/restrictions: The data are owned by TU Darmstadt. Requests to access these datasets should be directed to JS, josef.schlittenlacher@manchester.ac.uk.

## Ethics Statement

Ethical review and approval was not required for the study on human participants in accordance with the local legislation and institutional requirements. The patients/participants provided their written informed consent to participate in this study.

## Author Contributions

JS implemented the software to run the experiments, supervised the data collection, and analysed the data. JS and WE interpreted the results and wrote and revised the manuscript. Both authors contributed to the article and approved the submitted version.

## Conflict of Interest

The authors declare that the research was conducted in the absence of any commercial or financial relationships that could be construed as a potential conflict of interest.
